# Forest insects and climate change: long-term trends in herbivore damage

**DOI:** 10.1002/ece3.717

**Published:** 2013-09-30

**Authors:** Maartje J Klapwijk, György Csóka, Anikó Hirka, Christer Björkman

**Affiliations:** 1 Department of Ecology, Swedish University of Agricultural SciencesUppsala, Sweden; 2 Department of Forest Protection, Forest Research InstituteMátrafüred, Hungary

**Keywords:** Herbivory, Hungary, Lepidoptera, moths, precipitation, temperature, variability, weather

## Abstract

Long-term data sets, covering several decades, could help to reveal the effects of observed climate change on herbivore damage to plants. However, sufficiently long time series in ecology are scarce. The research presented here analyzes a long-term data set collected by the Hungarian Forest Research Institute over the period 1961–2009. The number of hectares with visible defoliation was estimated and documented for several forest insect pest species. This resulted in a unique time series that provides us with the opportunity to compare insect damage trends with trends in weather patterns. Data were analyzed for six lepidopteran species: *Thaumetopoea processionea*, *Tortrix viridana*, *Rhyacionia buoliana*, *Malacosoma neustria*, *Euproctis chrysorrhoea*, and *Lymantria dispar*. All these species exhibit outbreak dynamics in Hungary. Five of these species prefer deciduous tree species as their host plants, whereas *R. buoliana* is a specialist on *Pinus* spp. The data were analyzed using general linear models and generalized least squares regression in relation to mean monthly temperature and precipitation. Temperature increased considerably, especially over the last 25 years (+1.6°C), whereas precipitation exhibited no trend over the period. No change in weather variability over time was observed. There was increased damage caused by two species on deciduous trees. The area of damage attributed to *R. buoliana* decreased over the study period. There was no evidence of increased variability in damage. We conclude that species exhibiting a trend toward outbreak-level damage over a greater geographical area may be positively affected by changes in weather conditions coinciding with important life stages. Strong associations between the geographical extent of severe damage and monthly temperature and precipitation are difficult to confirm, studying the life-history traits of species could help to increase understanding of responses to climate change.

## Introduction

In recent decades, changes in weather patterns have been observed, the most pronounced being an increase in ambient temperatures (Solomon et al. 2007). In addition, there is previous research that has recorded changes in species ranges, phenology of life cycles, and interactions (Walther et al. 2002; Parmesan and Yohe 2003; Menzel et al. 2006; Visser and Both 2006; Walther 2010). Species range expansions and, to a lesser extent, retractions have been observed (Parmesan et al. 1999; Parmesan 2006) as well as changes in population dynamics in response to changing winter temperatures and snow cover (Ims and Fuglei 2005; Bale and Hayward 2010). Changes in outbreak patterns (Esper et al. 2007) and in outbreak range (Jepsen et al. 2008) have been attributed to climate warming. One example is the mountain pine beetle (*Dendroctonus ponderosae*) in North America, which has expanded its range as a result of increased minimum winter temperatures and has achieved higher densities as a result of increased survival and availability of even-aged host plant stands, resulting in unprecedented damage to forests in North America (Robertson et al. 2009; Cudmore et al. 2010). More often patterns of forest pest insect damage have a complex relationship with weather factors or associated changes.

Drought commonly increases herbivore damage (Csóka 1996, 1997; Jactel et al. 2012). Drought stress situations that have a positive effect on host plant quality for insects may arise in situations with high temperature and/or low precipitation (Koricheva et al. 1998). There is evidence that high levels of damage are related to unusual weather patterns (Martinat 1987 and references therein), but the patterns are inconsistent. Insects are ectotherms and could, therefore, be expected to respond strongly to changes in their external environment. Decreased development times increase the potential for multiple generations within the same growing season for multivoltine species as well as for species previously considered to be univoltine (Altermatt 2010; Pöyry et al. 2011). Other studies have found that increased temperatures lead to more fecund adults (Laws and Belovsky 2010). Because temperature often has a direct positive effect on insects in many of their life stages, it is readily concluded that insect populations will exhibit improved performance in a warmer climate. This simple causal linkage fuels the fear of increased damage by pest insects.

Researchers investigating latitudinal gradients showed greater herbivore damage at lower (warmer) latitudes (Adams and Zhang 2009), but other studies have failed to reveal similar herbivore damage patterns (Andrew and Hughes 2004; Sinclair and Hughes 2008). Causal links are unclear and subject of debate, but conclusive findings are lacking (Björkman et al. 2011). Thus, the current evidence seems to suggest no general conclusion concerning levels of herbivore damage in relation to increasing temperatures.

Potential effects of climate change on herbivore damage are not related only to direct effects of weather variables on insect herbivores, indirect effects also need to be considered (Klapwijk et al. 2012). Weather variables can affect insect larvae and the damage they do directly by influencing factors such as winter survival and metabolic rates. However, host plant quality and natural enemy pressure are affected by fluctuations in weather patterns as well, which, in turn, will affect the damage levels as well. In general, the effects of weather may contribute to and strengthen the effects of induced responses of the host tree to herbivore damage (Baltensweiler et al. 2008) and the effects of natural enemies, especially parasitoids, that will be observed in the next or subsequent generations (Liebhold et al. 2000; Klapwijk et al. 2012 and references therein). However, these responses might be asymmetrical (Berggren et al. 2009) resulting in increased predator efficiency as a result of increased temperatures (Kruse et al. 2008). The outcomes of all possible interactions between plants, herbivores, and their enemies in a food web are reflected in the levels of and fluctuations in herbivore damage.

Analysis using the volume of damaged wood (m^3^) removed from the forest as indicator of bark beetle damage (*Ips typographus*) showed drought to be one of the factors influencing levels of damage (Marini et al. 2012). They also found endogenous negative feedback with a 2-year lag, suggesting a potentially important role for natural enemies of *I. typographus* in forests in the Italian Alps. A major obstacle when studying the possible link between weather and dynamics is the lack of long-term data sets as most ecological data collections span 10–20 years. The difficulties are particularly problematic for outbreak species because the time frames of data collection would most likely encompass only one or two outbreaks providing meager data for analyses.

**Figure 1:**
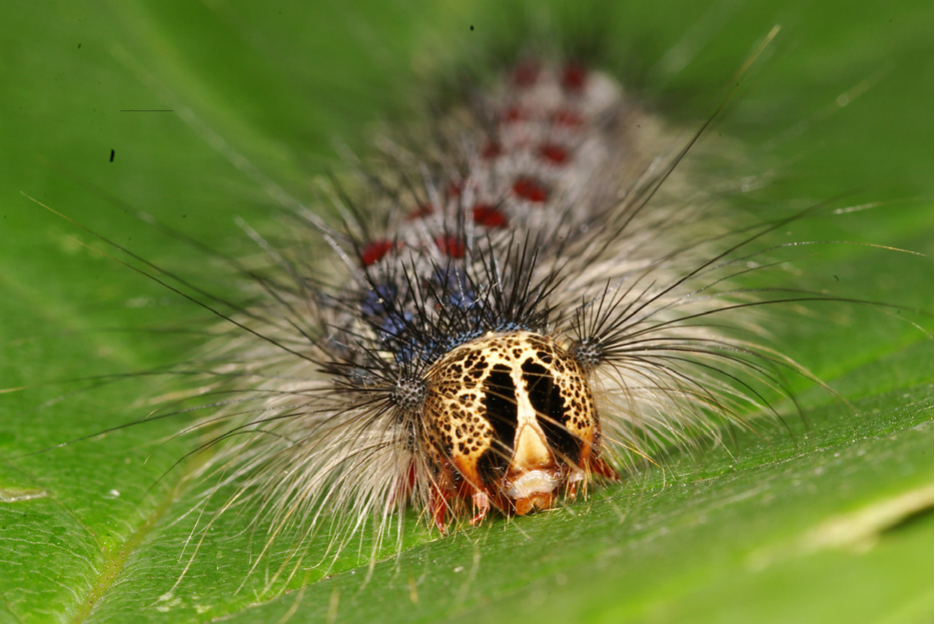
Larva of Gypsy moth (*Lymantria dispar*) the most notorious of the six outbreak species analyzed.

In this study, “area of damage” caused by herbivorous forest pest insects (i.e., the area of forest subjected to more than 20% defoliation) and associated year-to-year fluctuations are examined in relation to observed climate change. We have the unique opportunity to present damage data collected by the Hungarian Forest Research Institute from 1961 to 2009 for six forest insect species (Fig. 1). The damage of these six species could be contributed to specific species in the field whereas other data collected are grouped per family. We analyze the data for trends over time in observed damage area and for relations with temperature and precipitation. Also, we investigate potential changes in the amplitude of the fluctuations over time.

The aim of this study was to investigate whether there are relationships between herbivore damage and temperature or precipitation. The two questions we endeavor to answer are as follows:

- Are there trends in the area of insect damage and/or variability in insect damage during the period 1961–2009?
- How are weather variables in year (*t*) and year (*t* − 1) related to damage in year (*t*)?

An extensive damage area indicates widely distributed populations, and visible herbivore damage indicates high numbers of individuals. Fluctuations in damage area reflect population densities over a certain threshold over time and space. These fluctuations can be used to assess the relationship between the area of herbivore damage and weather conditions (Miller et al. 1989; Marini et al. 2012). The data set includes Lepidoptera species notorious for causing damage in Hungarian forests. These species cause high levels of damage at more or less regular intervals and are often referred to as “outbreak species” (Berryman 1987).

Outbreak species are often characterized by high potential population growth rates (Wallner 1987; Hunter 1991) resulting in the capacity for rapid population increases from 1 year to the next, often leading to high levels of herbivore damage. The population dynamics of species with high potential population growth rates are more vulnerable to fluctuations due to density-dependent regulation than species with low population growth rates (Turchin and Taylor 1992; Turchin 2003). Exogenous variables and endogenous negative feedback together could result in irregular and more or less cyclical patterns in population dynamics (Turchin 2003).

If changes in weather patterns were linked to the overall changes in climate, one would expect changes in insect population fluctuations over time. Theoretically, a possible mechanism behind amplified fluctuations could be increased realized population growth rates (Hassell et al. 1976). Such positive direct effects of climate warming have been found, for example, in the pine processionary moth (*Thaumetopoea pityocampa*) and the mountain pine beetle (*Dendroctonus ponderosae*) as a consequence of higher winter temperatures, increasing winter survival, and thus higher realized population growth rates, leading to greater damage (Battisti et al. 2005; Cudmore et al. 2010).

Positive indirect effects might be observed if density-dependent population regulation becomes weaker; this could potentially lead to similar variation around an increasing population mean. On the other hand, if natural enemy pressure becomes stronger or the potential number of individuals sustained in a population is reduced, the population will exhibit greater fluctuations. Higher frequency and severity of outbreaks lead to more damage, but it is still debatable whether or not global warming will result in a higher risk of insect outbreaks (Klapwijk et al. 2012 and reference therein). We found that even though we can observe changes over time, these changes are species specific. Also, there is an indication of a direct relationship between these changes and climatic variables, but the exact mechanisms are hard to identify.

## Material and Methods

### Data collection

**Figure 2:**
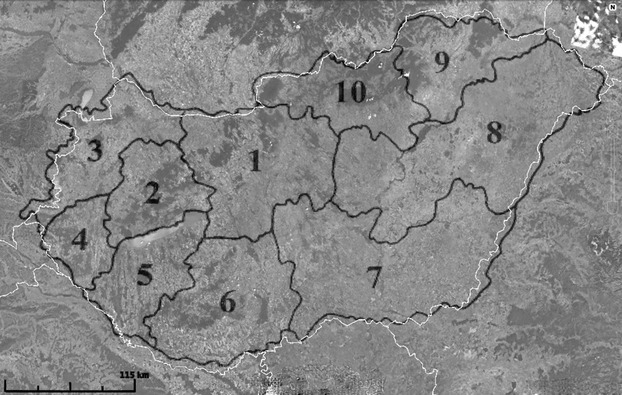
Map of Hungary with the forest insect damage regions shown (latitude 47–49°N longitude 16–23°E).

The total area of forest in Hungary is 1,832,000 ha; of which 1,320,000 ha are hardwood forests, 292,000 ha are softwood forests (*Populus*, *Salix*, etc.), and 220,000 ha are covered by coniferous tree species. Hungary can be divided into 10 different regions, belonging to the 10 directorates of the State Forest Service (Fig. 2). The damage data were collected separately each year from 10 different areas. Forest companies, private forest owners, and forest managers are obliged by law to report damage occurring in their forests four times a year, following detailed guidelines (Hirka and Csóka 2006). These reports are collected, checked, validated, and then summarized by the Department of Forest Protection of the Hungarian Forest Research Institute. The values analyzed refer to areas with defoliation exceeding 20% of all foliage, except for data pertaining to *Rhyacionia buoliana*, where it refers to an infestation rate exceeding 10% (of the total number of stems). In contrast to defoliation damage, *R. buoliana* damage is a long-lasting one (practically will last in the whole life of the stand). The distortion of the leading shoot has stronger and longer lasting influence on the single tree than most of the other damage forms.

For the purpose of this study, we used the full set of data on area damage per year for the whole of Hungary. Because the data were collected using a standardized method, the sources (i.e., observer) of error when assessing damaged forest area per region were not considered.

Weather data were collected by the Hungarian Weather Service (Országos Meteorológiai Szolgálat; http://www.met.hu/en/idojaras/) and provided mean monthly temperatures per weather station (13 in total). For the purpose of the data analyses, the monthly temperature and rainfall at each weather station was combined to give a country average and an annual country average. The mean yearly temperatures and precipitation were calculated on the basis of mean monthly temperature and mean monthly precipitation collected by the Hungarian Meteorological Institute. The weather data were grouped into 10-year periods for which the yearly mean and standard error were calculated in order to assess any potential change in variability between years within the 10-year time periods.

### Biology of the insect species

*Euproctis chrysorrhoea* L. (Lepidoptera, Lymantriidae), Brown-tail moth, is univoltine, lays eggs in July/August, and hatches in late August or early September. This species overwinters as larvae in nests made of leaves and silk spun together on branches in tree crowns. In spring, they commence feeding gregariously, mainly on *Quercus* spp., but will also feed on other deciduous trees, spinning silk webs at every fresh feeding site; later, they continue to feed alone. The pupae remain in their silken cocoons in May/early June. The adults fly at dusk from late May till July, August or September, depending on the site and the year.

*Lymantria dispar* L. (Lepidoptera, Lymantriidae), Gypsy moth, is univoltine; its larvae overwinter inside the eggs from August to April. The larval stage lasts from April to early July, approximately 8–12 weeks, depending on temperature. Pupae can be found from June until August in a silken cocoon among foliage. The adult male flies by day from June/July until early September, depending on the site and the year. The female does not fly and rarely moves far from the cocoon (Carter 1984). The main host trees are *Quercus* spp., but the species can be found on a whole range of deciduous trees.

*Tortrix viridana* L. (Lepidoptera, Tortricidae), Green oak leaf roller, is a univoltine species, which overwinters in the egg stage from June to April/May and hatches in April or early May. The larvae feed from April until May/June, first on buds and, thereafter, rolled into a leaf. They are polyphagous, but the main host trees are *Quercus* spp. The larvae pupate in May/early June in a rolled or folded leaf. The adults fly at night from late May to early July.

*Rhyacionia buoliana* (Schiffermüller 1775; Lepidoptera, Tortricidae), European pine shoot moth, is univoltine, overwinters as third-instar larvae and breaks diapause at the beginning of April. The larvae pupate in June and emerge in July or August, after which they immediately lay eggs on pine shoots. The eggs hatch in August, and the larvae start feeding on the shoots. Third-instar larvae bore into the shoot and overwinter there.

*Thaumetopoea processionea* L. (Lepidoptera, Notodontidae), Oak Processionary moth, is a univoltine species that overwinters as eggs, in batches of 100–200; its larvae overwinter inside the eggs as neonates (Wagenhoff and Veit 2011). From late April to June, the larvae live in a communal nest that they leave at night to feed on the foliage of *Quercus* spp. (mainly *Q. cerris* in Hungary). The larvae pupate in cocoons within the communal web and emerge from July to August, flying at night.

*Malacosoma neustria* L. (Lepidoptera, Lasiocampidae), Lackey moth, is univoltine and overwinters as pharate larvae inside the egg from August to the following April; eggs are laid in groups of 100–250. Larvae feed gregariously on *Quercus* spp. or other deciduous trees from April until May/June in a large communal web or tent. The larvae pupate in May/July in silken cocoons spun between leaves, on the trunk or on the ground. The adults fly at night from late May/June until August, depending on the site and year.

For all these species, Hungary is in the center of their European range distribution (Mészáros and Szabóky 2005, 2012). All species, except *T. viridana* and *R. buoliana*, feed gregariously throughout their life time or for a large part of their time as larvae. *Lymantria dispar* is the only species with low mobility females. All species, except *E. chrysorrhoea* and *R. buoliana*, overwinter as eggs and feed in spring. However, the larvae of *L. dispar* and *M. neustria* overwinter inside the egg as neonate larvae. (Table 1).

**Table 1:** The timing of the life cycles of the species studied

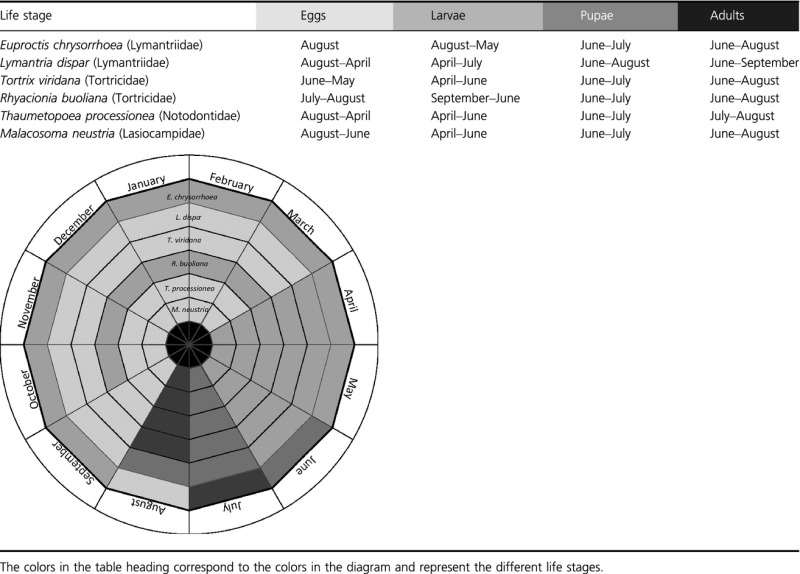

### Data analyses

The yearly average temperature and rainfall were calculated and used to calculate means and standard errors in the following periods 1961–1970, 1971–1980, 1981–1990, 1991–2000, and 2001–2009 in order to assess changes in variance. Generalized additive models were used to investigate temporal trends in the yearly and monthly average temperature and rainfall. To analyze the relationship between damage and average monthly precipitation and temperature, the data were appropriately aligned so that the influence of weather after the larval period (e.g., pupal stage, adult stage and egg stage) would be related to the damage in the following year. This leads to the use of damage data collected from 1963 to 2009, direct temperature and precipitation data from, respectively, 1962 to 2008 (aligned to the pupal, adult, egg/larval stages) and 1963 to 2009 (aligned to the egg/larval stage), and lagged temperature data from 1961 to 2007 (aligned to the pupal, adult, egg/larval stages) and 1962 to 2008 (aligned to the egg/larval stage). Therefore, the total analysis period was 47 years, although it was only 46 years for *M. neustria* as a result of missing values. The damage data was log_e_ transformed prior to analyses.

The analyses were carried out in the following order: first, the data were checked for periodicity, after that general trends in the damage data were analyzed, and before investigating the relationship of the damage with temperature and precipitation trends these variables were checked for patterns and trends.

### Trends over time

A General Additive Model (GAM) was used to estimate the trend over time for mean yearly temperatures, precipitation, and for damage data (hectares). These models allow for the inclusion of a nonparametric smoothing and will fit a regression spline to the data, allowing for nonlinear relationships (Wood 2006; Zuur et al. 2007). The model returns estimated degree of freedom (edf), edf = 1 represents a linear relationship, edf > 1 represents a nonlinear relationship with edf = 4 approaching a third level polynomial relationship (Zuur et al. 2007). The same procedure was followed for the damage caused by the different species over time and for average yearly precipitation and temperature.

These models and all the other models used were inspected for the presence of heteroscedasticity in the residuals. Any patterns of increasing variance (increasing damage variability over time) were detected by plotting the expected values of the model against the residuals.

### Damage area in relation to monthly temperature and precipitation

For all species, preliminary analyses were conducted using generalized additive mixed models (GAMM; Wood 2006; Zuur et al. 2009) for temperature and precipitation in each month separately. Such models are the ‘additive’ equivalent of generalized linear mixed effect models (Zuur et al. 2007, 2009) This class of models allows for the combination of fixed parametric effects and nonparametric smoothers in a single model. The method has been increasingly applied to ecological problems (Pierce et al. 2007 and others).

In each model, an autocorrelation structure was included to account for temporal autocorrelation in the time series. Model comparison, based on the null model, was used to estimate the most appropriate autoregressive function for the residuals; we tested an autoregressive model of order 1 (white noise) and the autoregressive model average function (ARMA). The intercept-only model for *L. dispar* showed a better fit with ARMA (*p* = 2, *q* = 1). When weather variables were added to the model the model did not improve, and no variables were found to be significant. Therefore, only the results of the model with the AR(1) function and significant weather variables are presented.

An autoregressive model of order 1 was chosen as the error structure, meaning that the residuals at time *t* are modeled as a function of time *t* − 1 along with white noise (eq. 1; Zuur et al. 2009); this construction included the variable ‘year’ in the model.


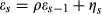
(1)

After the initial data exploration, the significant variables were combined in a full model and depending on whether or not the relationship was linear or nonlinear analysis was carried out with GAMM (nonlinear) or a generalized least square regression (linear). The relationship between temperature and precipitation in the winter months (December–February) was included to investigate the potential relationship between insulation by snow and increased survival (Bale and Hayward 2010). No such significant additive relationship was found and hence the results are not presented.

The same procedure was repeated using temperature at lag 1 to investigate whether there were delayed effects of temperature. For precipitation, only direct effects on damage levels were taken into account following the same procedure.

No other interactions were included in the model although numerous potential combinations could have biological significance. Including too many interactions would compromise the reliability of the model output and the residual degrees of freedom would be strongly reduced. Another reason is that, although the time series is long from an ecological perspective, it only provides 48 df (*n* = 49) and the analysis consumes 12 df for temperature and 12 df for precipitation. The inclusion of multiple interactions would have led to low residual degrees of freedom in relation to the total degrees of freedom, leading to an increased probability of a type II error.

After composition of the full model, the variables were removed from the model according to the principles of marginality until the model contained only parameters significant at the 5% level. The total variance in the model was estimated by fitting the null model (the intercept only model) to extract the residual standard error and calculate the error variance. The following function was used to calculate the proportion of explained variation:


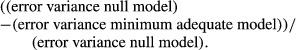


## Results

### Periodicity in the time series

**Figure 3:**
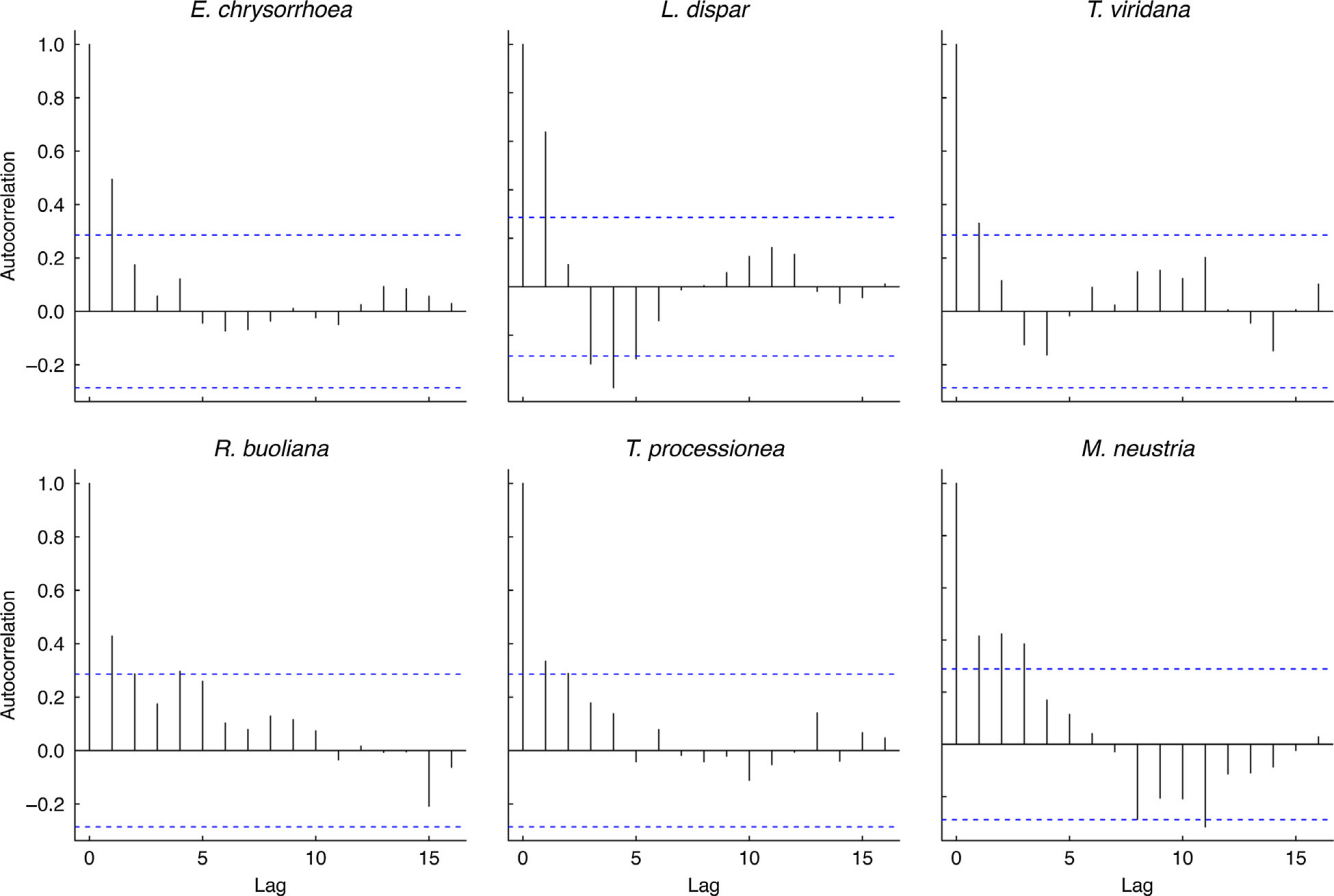
The autocorrelation functions for each forest insect species. Both *Euproctis chrysorrhoea* and *Tortrix viridana* have nonstationary times series with suggested cycles. *Lymantria dispar* has a cyclic time series with periodic cycles of about 10 years. Both *Rhyacionia buoliana* and *Thaumetopoea processionea* have nonstationary time series without periodicity. *Malasocoma neustria* shows no periodicity or, possibly, cycles of 20 years that cannot be confirmed by this data.

*Lymantria dispar* is the only species showing clear periodicity over time (Table 2; Fig. 3). Analysis using autocorrelation and partial autocorrelation functions indicates a cyclicity of roughly 10 years and no tendency toward change over time. The relatively high φ-value indicates a strong relationship between the extents of damage in consecutive years (Table 2). The low strength of the autocorrelation (φ-value; Table 2) for the other species indicates very weak or a lack of periodicity. The autocorrelation functions for *E. chrysorrhoea* and *T. viridana* do suggest the possibility of some periodicity in their time series. Both *R. buoliana* and *T. processionea* had a nonstationary mean and no periodicity. *Malacosoma neustria* either has a nonstationary time series or, perhaps, a very long cycle.

**Table 2:** The results of the GAMM/GLS analyses

Species	Parametric	Smoother	ndf, ddf; *F*/*t*-value; *P*-value	Estimate (±SE mean)	RSE	φ	95% CI φ
*Euproctis chrysorrhoea*	Intercept		df = 1,44; *F* = 321.5, *P* < 0.001	−6.19 ± 3.96	1.45	0.49	(0.17; 0.72)
	July (*t*)		df = 1,44, *F* = 4.06, *P* = 0.05	0.30 ± 0.14			
	June (*t*; lag 1)		df = 1,44, *F* = 6.09, *P* = 0.02	0.33 ± 0.13			
*Lymantria dispar*	Intercept		df = 1,43, *F* = 266.64, *P* < 0.001	16.38 ± 1.66	1.28	0.73	(0.48; 0.88)
	October (*t*)		df = 1,43, *F* = 7.70, *P* = 0.01	−0.41 ± 0.09			
	March (*t*; lag 1)		df = 1,43, *F* = 8.37, *P* = 0.01	−0.15 ± 0.06			
	October (*t*; lag 1)		df = 1,43, *F* = 10.64, P = 0.002	−0.31 ± 0.10			
*Tortrix viridana*	Intercept		df = 1,43, *t* = 1.54, *P* = 0.14	4.32 ± 2.80	1.22	0.53	(0.21; 0.74)
		June (*t*)	edf^1^ = 3.95, *F* = 5.70, *P* = 0.001	1.70 ± 0.83			
	July (*t*; lag 1)		df = 1,43, *t* = 2.58, *P* = 0.01	0.35 ± 0.14			
	September (*t*; lag 1)		df = 1,43, *t* = −2.96, *P* = 0.005	−0.32 ± 0.11			
*Rhyacionia buoliana*	Intercept		df = 1,44, *F* = 635.53, *P* < 0.001	9.45 ± 1.14	1.09	0.41	(0.06; 0.66)
	January (*t*)		df = 1,44, *F* = 8.43, *P* < 0.006	−0.16 ± 0.06			
	October (*t*)		df = 1,44, *F* = 8.80, *P* < 0.005	−0.31 ± 0.11			
*Thaumetopoea processionea*	Intercept		df = 1,42, *F* = 144.24, *P* < 0.0001	−8.02 ± 3.75	1.78	0.54	(0.19; 0.77)
	March (*t*)		df = 1,42, *F* = 7.07, *P* = 0.011	−0.24 ± 0.10			
	April (*t*)		df = 1,42, *F* = 10.25, *P* = 0.003	0.40 ± 0.15			
	July (*t*)		df = 1,42, *F* = 4.62, *P* = 0.037	0.44 ± 0.17			
	May (*p*)		df = 1,42, *F* = 9.90, *P* = 0.003	0.02 ± 0.007			
*Malacosoma neustria*	Intercept		df = 1,44, *F* = 198.63, *P* < 0.0001	5.38 ± 0.37	1.52	0.44	(0.16; 0.66)
	February (*t*; lag 1)		df = 1,44, *F* = 9.12, *P* = 0.004	−0.21 ± 0.07			

When smoothing parameters are reported the analysis used was generalized additive mixed models (GAMM). If only parametric parameters were included generalized least square (GLS) was used for the analysis. In both models, potential autocorrelation in the residuals was dealt with by including a random walk correlation structure. If the variable was monthly temperature, the month is followed by (*t*); in the case of precipitation, the month is followed by (*p*). Lagged effects are annotated (lag 1). GAMM returns estimated degrees of freedom (edf), edf = 1 represents a linear relationship, edf > 1 represents a nonlinear relationship with edf = 4 approaching a third level polynomial relationship (Zuur et al. 2007).

### Changes in extent and/or variability in damage

**Figure 4:**
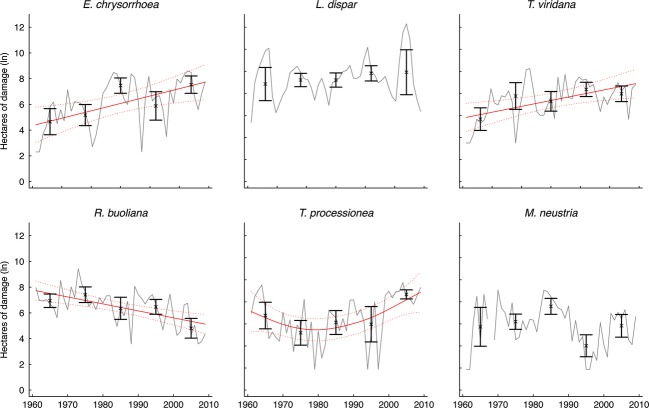
Trends over time for the damage caused by the different forest insect species using either general additive models or general least squares regression. For every period of 10 years, the mean and 95% confidence interval were calculated and these are presented in the graphs.

The untransformed damage data are summarized in Table 3 and the coefficient of variation is presented for each species to illustrate the total variability within the data set. Analyzing the damage trends over the period of observation for *T. viridana* (0.77 ± 0.46 [estimate ± standard error], *F* = 7.92, *P* = 0.007) and *E. chrysorrhoea* (0.98 ± 0.34, *F* = 8.2, *P* = 0.06) damage area shows a significant linear increase over time (Fig. 4). *Thaumetopoea processionea* (edf = 2.27, *F* = 4.55, *P* = 0.013) damage area exhibited a U-shaped trend over time (Fig. 4). Both *M. neustria* (*F* = 0.048, *P* = 0.83) and *L. dispar* (*F* = 0.38, *P* = 0.54) did not exhibit a significant trend in damage area over time (Fig. 4). *Rhyacionia buoliana* (−0.77 ± 0.19, *F* = 16.86, *P* < 0.001) is the only species for which the damage area decreased linearly over time (Fig. 4). None of the species exhibited increasing variability in damage over time. Comparison of the fitted values versus the residual values of the regression of damage area over time for each separate model revealed no patterns with respect to the variance. No general trends linking average yearly temperature and precipitation to damage area were found for any of the species and no clear pattern in residuals (heteroscedasticity) could be detected as a signal of increasing variability.

**Table 3:** Summary of the raw data for the damage area (hectares) for each species, minimum, mean and maximum, and the calculated coefficient of variation (CV)

		Extent of damage (ha)		
		
	Mean	Minimum	Maximum	CV
*Euproctis chrysorrhoea*	1259	10	5452	1.23
*Lymantria dispar*	12,703	100	212,177	2.70
*Tortrix viridana*	1208	20	6561	1.19
*Rhyacionia buoliana*	1280	36	12,461	1.51
*Thaumetopoea processionea*	778	5	4270	1.27
*Malacosoma neustria*	447	5	2389	1.39

### Changes in temperature and precipitation over time

**Figure 5:**
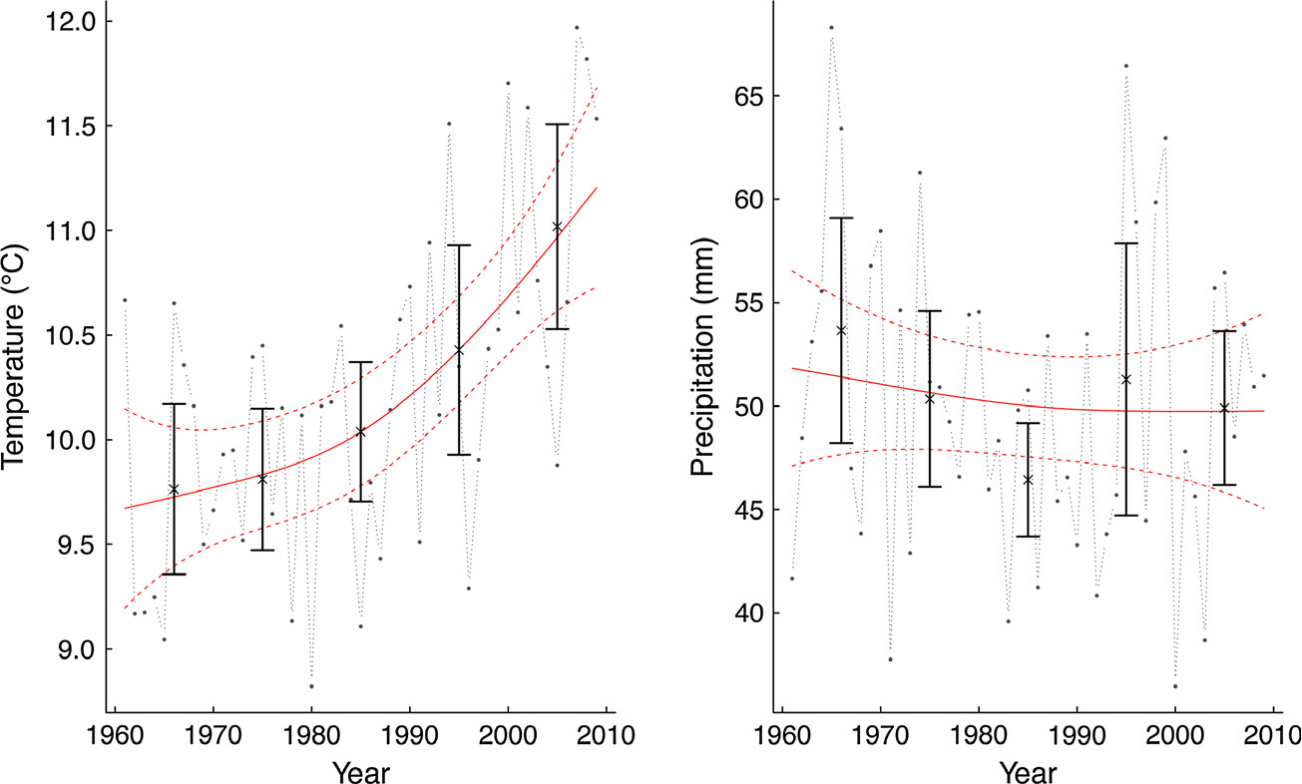
Trends in temperature and precipitation in Hungary from 1961 to 2009 (and the 95% confidence intervals). The trend for temperature is significant (edf = 1.94, *F* = 11.73, *P* < 0.001). The variation in mean yearly temperature has not increased during the time period. Precipitation has not increased over time and variability between years has also not increased over time. For every period of 10 years, the mean and 95% confidence interval were calculated and these are presented in the graphs.

The mean annual temperature exhibited an increase from 9.6 to 11.2°C (+1.6°C) during the 49-year study period, but the increase accelerated after 1985 (Fig. 5A). There was no significant change in precipitation over the 49 years (Fig. 5B). The correlation of temperature with precipitation in the same month over time was generally low (r ranged from −0.51 to 0.21) and the correlations between months, for both temperature and precipitation, were generally weak, with r-values ranging from −0.43 to 0.45. Analyses of the months separately showed that significant increases in mean monthly temperatures occurred for January, April, May, June, July, and August, and that the relationships have a tendency toward nonlinearity, as shown by the analyses using GAMs (Table 4). Analysis showed no change in mean monthly precipitation for the individual months (Table 4). The residuals of the models were examined for inequality of variances, but no heteroscedasticity was detected.

**Table 4:** The results of the general additive models for average monthly temperatures and average monthly precipitation over time

Temperature (°C)						Precipitation (mm)			
	
Month	Intercept	*t*-value	edf	*F*-value	Adjusted *R*^2^	Intercept	*t*-value	edf	*F*-value
January	−1.19 ± 033	−3.56***	1	8.43**	0.13	32.32 ± 2.22	14.95***	1	0.97
February	−0.97 ± 0.40	−2.38*	1.54	0.98		30.47 ± 2.57	11.85***	1.54	0.40
March	5.34 ± 0.31	17.38***	1	2		35.74 ± 2.24	15.98***	1.28	0.84
April	10.77 ± 0.18	60.63***	3.34	4.78**	0.28	46.61 ± 2.54	18.37***	3.63	0.80
May	15.71 ± 0.20	78***	1	8.04**	0.13	61.88 ± 3.52	17.61***	1	0.34
June	18.83 ± 0.17	111***	2.10	3.99*	0.18	75.60 ± 4.00	18.88***	1	0.19
July	20.57 ± 0.17	121.4***	1.43	7.40**	0.23	67.66 ± 3.79	17.84***	1.67	0.14
August	20.08 ± 0.19	106.1***	2.20	5.07**	0.22	64.30 ± 4.17	15.41***	1	0.06
September	15.78 ± 0.20	77.42***	1	0.16		52.06 ± 4.12	12.63***	1	1.01
October	10.53 ± 0.20	52.89***	1.90	1.63		42.62 ± 4.09	10.43***	1	0.21
November	4.81 ± 0.27	18.14***	2.12	2.49		52.03 ± 3.69	14.09***	2.49	1.84
December	0.18 ± 0.26	0.67	3.01	1.66		42.70 ± 2.76	15.45***	1	0.07

The *t*-value corresponds to the intercept and the *F*-value corresponds to the smoothing parameter. The significance levels are indicated with asterisks (‘***’, <0.0001; ‘**’, 0.001; ‘*’, 0.01; ‘.’, 0.05). For the significant smoothers, the Adjusted *R*^2^ is given as an indication of explained variation. Generalized additive mixed models returns estimated degrees of freedom (edf), edf = 1 represents a linear relationship, edf > 1 represents a nonlinear relationship with edf = 4 approaching a third level polynomial relationship (Zuur et al. 2007).

### Relationship of damage with monthly temperatures (direct and lag 1) and precipitation?

**Figure 6:**
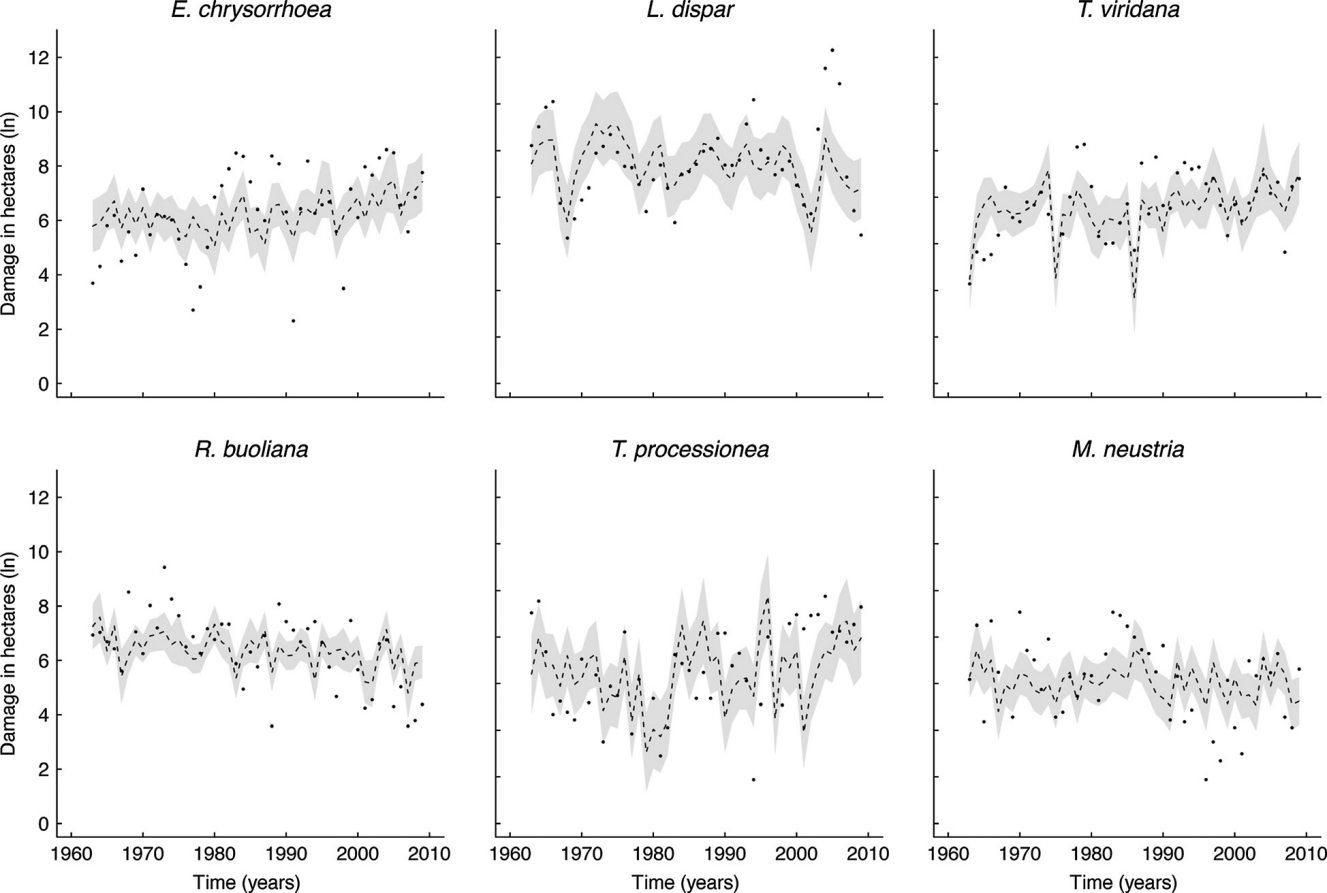
The fitted values of the model for each forest insect species (solid lines) ± 95% confidence interval (dashed lines) over time. The model for *Malacosoma neustria* explains the least total variation (14%) whereas the model for *Tortrix viridana* explains the largest amount of the total variation, 37%, *Rhyacionia buoliana* comes second with 30% explained variation. The models for *Euproctis chrysorrhoea*, *Lymantria dispar*, and *Thaumetopoea processionea* explain 24%, 25%, and 22%, respectively

*Euproctis chrysorrhoea* damage area exhibits a positive correlation with July temperatures and there is a positive relationship between damage area and temperatures in June in the previous year. The correlation between *t* and *t* − 1 is not very strong, but it is significant as zero falls outside the 95% confidence intervals (95% CI). The model explains 24% of the total variation (Table 2; Fig. 6).

There are negative relationships between *L. dispar* damage area and October temperatures and between damage and March and October temperatures in the previous year. The correlation between *t* and *t* − 1 is strong and significant as zero falls outside the 95% CI. The model explains 25% of the total variation in *L. dispar* damage area.

The damage area of *R. buoliana* decreases with increasing January and October temperatures, explaining 30% of the total variation. The correlation between *t* and *t* − 1 is not very strong, but is significant on the basis of the 95% CI.

*Tortrix viridana* is the only species with a nonlinear relationship within the model. The strength of the response to June temperatures accelerates at higher temperatures. There was a positive linear relationship between damage area and July and September temperatures in the previous year. The model explains 37% of the variation. There is a weak correlation between *t* and *t* − 1, but this is significant on the basis of the 95% CI.

There is a negative linear relationship between *M. neustria* damage area and February temperatures in the previous year, however, only 14% of the total variation is explained by this model. The correlation between damage area at *t* and *t* − 1 is fairly weak but significant.

The damage area of *T. processionea* is negatively correlated with March temperatures. Positive relationships are found for April and July temperatures and for precipitation in May. The model explains 22% of the total variation in damage area. The correlation between years is relatively weak but significant on the basis of the 95% CI.

## Discussion

This study is one of only a few to investigate the damage caused by several forest pest insects over a relatively long time period. The general finding is that there is no single trend for all six investigated species. Recent studies suggest that damage by herbivores should increase with increasing temperature (Adams and Zhang 2009), but since most of these studies use temperature gradients involving altitude or latitude as proxies for climate change they are not directly applicable to temporal changes. The analyses of temperature and precipitation data in this study showed that mean yearly temperature has been steadily rising since roughly 1985 (Fig 2A). This steady rise does not directly relate to changes in damage area over the same time span. Precipitation patterns seem not to have changed in that period and also do not show a direct relationship with damage area. This lack of common trends over time indicates the need for more detailed analyses.

The damage data used in the time series (area of damage in hectares) is an indirect measure of population dynamics. The damage recorded is defoliation over 20% (or 10% infestation for *R. buoliana*) and thus actually reflects the outbreak area of a species at any given time in Hungary. Detailed density sampling would not have provided this type of large-scale information even though it might have given more insight into the actual mechanisms behind the population dynamics observed. The data, as collected and analyzed, give us an insight into the influence of weather variables on the extent of outbreak areas of certain species and reveals several interesting temporal patterns. Changes in damage levels and damage patterns over time could, potentially, be attributed to changes in the area of forest available for these insects. However, over the years examined, the area of forest has increased in Hungary over the study period by planting *Robinia* spp. stands, the forest area with the preferred tree species for the insects discussed in this study remained unchanged (G. Csóka, pers. comm.). The only observed change is the age structure of the pine forest, the influence of this change in age structure on *R. buoliana* was investigated for potential effects, but no relationship with the pattern of decreasing damage was found.

Our analyses led us to a number of conclusions. First, no obvious changes in the variability of outbreak area were recorded for any of the species during the data collection period. This means that the outbreaks are not spreading over large areas any more often than they were when data collection began. Second, even though the timing of their larval period is similar for the species examined, different trends in their long-term dynamics were observed. This indicates that changes in population densities do not depend only on direct effects on larval development and performance. Interestingly, both species with increasing trends in damage area over time have low CV's compared with the other species (Table 3). Unfortunately a quantitative comparison was not possible because of limited replication. Third, the observed increasing trends in damage for some species are not related to the overall increasing yearly mean temperatures, implying that the effect of changes in weather is operating during certain sensitive stages that might be species specific. Last, the two species with the highest coefficient of variation, *L. dispar* and *M. neustria* (Table 3), do not show an increasing trend. Interestingly, the damage caused by *R. buoliana*, which also has a high CV, actually reduced over the observation period (Fig. 4).

Our results indicate that there are direct as well as indirect effects of temperature on insect performance, potentially mediated by the host plant or natural enemies. Of all species, only *T. processionea* larvae seem to be affected by temperature during the larval growth period (temperatures in March and April). The negative relationship between March temperatures and subsequent outbreaks could be because high March temperatures cause hatching to be asynchronous with leaf flush; neonate larvae can survive some starvation, but if the period is too long it will have a negative effect on survival (Meurisse et al. 2012). However, if the high temperatures are a month later in April, they have a positive effect on damage area, indicating a positive effect on survival and feeding rates (Wagenhoff and Veit 2011). *Lymantria dispar* appears to be affected by March temperatures but with a delayed effect on damage in the subsequent year. Several species seem to be affected by July temperatures; *E. chrysorrhoea* and *T. processionea* in the same generation and *T. viridana* in the next generation. In July, these species are either in their pupal or adult stage and the direct effect on damage area could be the result of positive effects of temperature on dispersal, extending the damage area. The effect of dispersal can be observed in the same generation when the number of individuals dispersing is high or it can be seen in the following generation if the numbers are limited or the dispersed females have low fecundity, but their offspring are very successful in the newly colonized, previously undefoliated areas. Successful progeny of dispersed females, that is, a delayed effect of dispersal, could occur when dispersing individuals have low fecundity, a common feature in Lepidoptera (Saastamoinen et al. 2010). For both *R. buoliana* and *L. dispar*, temperatures in October have a negative relationship with damage area. This could indicate that warm Octobers lead to larvae (neonate larvae in the case of *L. dispar*) that are poorly prepared for overwintering, potentially because they use too much resource (Han and Bauce 1998 cited from Bale and Hayward 2010) or (perhaps more likely for *L. dispar* but also possible for *R. buoliana*) the extra heat provides parasitoids with a longer window of opportunity for attack and is associated with higher attack rates (Dhillon and Sharma 2009), reducing damage area in the next generation. For *R. buoliana* we identified a negative relationship between January temperatures and damage area; perhaps higher January temperatures keep metabolism up through winter, leading to individuals in poor condition when temperatures increase in spring (Hahn and Denlinger 2007). The lagged negative effect of September temperatures on *T. viridana* damage area is not straightforward to interpret. Effects on egg/larval survival should be observed in the damage caused by the generation in the following year. Potentially there is an effect of temperature on natural enemy survival, for example parasitoids; such increased parasitism success would only be observed in the herbivores 2 years later (Liebhold et al. 2000).

Using minimum and maximum temperatures might have led to clearer patterns in the data. However, mean monthly temperatures do reflect the minima and maxima to a certain extent; therefore, using these data in the analyses should have revealed the most important patterns of the relationship between temperature and area of damage for these forest insect Lepidoptera.

Certain outbreak species, like *L. dispar*, are known to have strong cyclical patterns of outbreaks (Turchin and Taylor 1992). Our analysis confirms this for the *L. dispar* damage area in Hungary. This cyclicity is often attributed to regulation by natural enemies based on theoretical models and some empirical data. The population cycles of *L. dispar* fit a second-order autoregressive model with a moving average. Including weather variables in this model does not add explanatory value. This could indicate that the population processes affecting *L. dispar* dynamics in Hungary are relatively insensitive to weather conditions. Alternatively, the processes may be sensitive to weather, as shown by the first-order regressive model with the weather variables included; this generates the type of pattern described by an ARMA model. However, it is unlikely that the population dynamics of *L. dispar* are independent of weather influences, even though previous studies have also suggested that the drivers of the cycles are endogenous (Liebhold et al. 2000). *Euproctis chrysorrhoea* and *T. viridana* show some indication of periodicity, but the noise in the data prevents the detection of true cycles over time. Potentially, the dynamics of the latter two species are not solely based on interactions with natural enemies or bottom-up control; the high percentages explained by the model (Table 4) support the relative importance of exogenous variation (e.g., weather fluctuations).

For most species, potential and detected cycles span more than about 10 years. This means that potential changes in periodicity are difficult to detect since a 49-year time series only covers four complete cycles at most. The potential cycles of *M. neustria* would span a period of more than 20 years, so this data set only covers, perhaps, one cycle. In summary, the actual frequency of peaks in damage area has not changed for any of the species. The potential and detected presence of 10-year cycles in the damage was also the reason to divide the damage in 10-year periods for the analyses of changes in the magnitude of variation over time. Approaching the variability in this manner the risk of unevenly divided peaks in the damage is brought to a minimum and the actual effect of higher amplitude of changes will be analyzed.

As discussed in the introduction, weather can have a great influence on many insect traits, resulting in observed changes in insect emergence and development times recorded as being a result of climatic change (Walther et al. 2002; Parmesan and Yohe 2003; Parmesan 2006). According to our analyses, temperature changes affect the damage caused by insect herbivores more strongly than precipitation. Our study does show that not all species are affected by climate change and that the species with a relatively high CV (indicating high rates of population change) will not respond in a similar manner to species with a low CV (indicating low rates of population change). Potentially, species with high population growth rates have population dynamics that are ruled more by endogenous feedback than by exogenous feedback; our results for *L. dispar* and *M. neustria* indicate that the feedback does not change. However, for *E. chrysorrhoea*, *T. viridana*, and *T. processionea*, it seems that the changes in exogenous feedback allow them to cause more than 20% damage to a greater area of forest. The observed reduction in *R. buoliana* damage area strengthens the finding of earlier research that autumn temperatures can be important for overwintering success, and that there is an additive effect of January temperatures (West 1936; Bogenschütz 1976). However, the observed relationships between damage and weather are not always intuitive and therefore highlight the need for a better understanding of the interactions between temperature, precipitation, insect physiology, and ultimately their effects on population dynamics.

## References

[b1] Adams JM, Zhang YJ (2009). Is there more insect folivory in warmer temperate climates? A latitudinal comparison of insect folivory in eastern North America. J. Ecol.

[b2] Altermatt F (2010). Climatic warming increases voltinism in European butterflies and moths. Proc. R. Soc. Lond. B. Biol. Sci.

[b3] Andrew NR, Hughes L (2004). Species diversity and structure of phytophagous beetle assemblages along a latitudinal gradient: predicting the potential impacts of climate change. Ecol. Entomol.

[b4] Bale JS, Hayward SAL (2010). Insect overwintering in a changing climate. J. Exp. Biol.

[b5] Baltensweiler W, Weber UM, Cherubini P (2008). Tracing the influence of larch-bud-moth insect outbreaks and weather conditions on larch tree-ring growth in Engadine (Switzerland). Oikos.

[b6] Battisti A, Stastny M, Netherer S, Robinet C, Schopf A, Roques A (2005). Expansion of geographic range in the pine processionary moth caused by increased winter temperatures. Ecol. Appl.

[b7] Berggren Å, Björkman C, Bylund H, Ayres MP (2009). The distribution and abundance of animal populations in a climate of uncertainty. Oikos.

[b8] Berryman AA, Barbosa P, Schultz JC (1987). The theory and classifications of outbreaks. Insect outbreaks.

[b9] Björkman C, Berggren A, Bylund H (2011). Causes behind insect folivory patterns in latitudinal gradients. J. Ecol.

[b10] Bogenschütz H (1976). Studies on the influence of temperature on the development of *Rhyacionia-buoliana* (Lepidoptera; Tortricidae). Z. Pflanzenkr. Pflanzenschutz.

[b11] Carter DJ (1984). Pest lepidoptera of Europe; with special reference to the British Isles.

[b12] Csóka G (1996). Aszályos évek- fokozódó rovarkárok erdeinkben (Years of drought – increasing damage by forest insects). Növényvédelem.

[b13] Csóka G (1997). Increased insect damage in Hungarian forests under drought impact. Biologia.

[b14] Cudmore TJ, Björklund N, Carroll AL, Lindgren BS (2010). Climate change and range expansion of an aggressive bark beetle: evidence of higher beetle reproduction in naive host tree populations. J. Appl. Ecol.

[b15] Dhillon MK, Sharma HC (2009). Temperature influences the performance and effectiveness of field and laboratory strains of the ichneumonid parasitoid, *Campoletis chlorideae*. Biocontrol.

[b16] Esper J, Büntgen U, Frank DC, Nievergelt D, Liebhold A (2007). 1200 years of regular outbreaks in alpine insects. Proc. R. Soc. Lond. B. Biol. Sci.

[b17] Hahn DA, Denlinger DL (2007). Meeting the energetic demands of insect diapause: nutrient storage and utilization. J. Insect Physiol.

[b18] Han EN, Bauce E (1998). Timing of diapause initiation, metabolic changes and overwintering survival of the spruce budworm, *Choristoneura fumiferana*. Ecol. Entomol.

[b19] Hassell MP, Lawton JH, May RM (1976). Patterns of dynamical behaviour in single-species populations. J. Anim. Ecol.

[b20] Hirka A, Csóka G (2006). Képes útmutató és kódjegyzék az erdővédelmi jelzőlapok kitöltéséhez (Guidelines and code list for forest damagage reports).

[b21] Hunter AF (1991). Traits that distinguish outbreaking and nonoutbreaking macrolepidoptera feeding on northern hardwood trees. Oikos.

[b22] Ims RA, Fuglei E (2005). Trophic interaction cycles in tundra ecosystems and the impact of climate change. Bioscience.

[b23] Jactel H, Petit J, Desprez-Loustau M-L, Delzon S, Piou D, Battisti A (2012). Drought effects on damage by forest insects and pathogens: a meta-analysis. Glob. Change Biol.

[b24] Jepsen JU, Hagen SB, Ims RA, Yoccoz NG (2008). Climate change and outbreaks of the geometrids *Operophtera brumata* and *Epirrita autumnata* in subarctic birch forest: evidence of a recent outbreak range expansion. J. Anim. Ecol.

[b25] Klapwijk MJ, Battisti A, Ayres MP, Larsson S, Barbosa P, Schultz JC, Letourneau D (2012). Assessing the impact of climate change on outbreak potential. Insect outbreaks revisited.

[b26] Koricheva J, Larsson S, Haukioja E (1998). Insect performance on experimentally stressed woody plants: a meta-analysis. Annu. Rev. Entomol.

[b27] Kruse PD, Toft S, Sunderland KD (2008). Temperature and prey capture: opposite relationships in two predator taxa. Ecol. Entomol.

[b28] Laws AN, Belovsky GE (2010). How will species respond to climate change? Examining the effects of temperature and population density on an herbivorous insect. Environ. Entomol.

[b29] Liebhold A, Elkinton J, Williams D, Muzika RM (2000). What causes outbreaks of the gypsy moth in North America?. Popul. Ecol.

[b30] Marini L, Ayres M, Battisti A, Faccoli M (2012). Climate affects severity and altitudinal distribution of outbreaks in an eruptive bark beetle. Clim. Change.

[b31] Martinat PJ, Barbosa P, Schultz JC (1987). The role of climatic variation and weather on forest insect outbreaks. Insect outbreaks.

[b32] Menzel A, Sparks TH, Estrella N, Koch E, Aasa A, Ahas R (2006). European phenological response to climate change matches the warming pattern. Glob. Change Biol.

[b33] Mészáros Z, Szabóky C (2005). A magyarországi nagylepkék gyakorlati albuma (Practical album of the Hungarian Macrolepidoptera).

[b34] Mészáros Z, Szabóky C (2012). A magyarországi nagylepkék gyakorlati albuma (Practical album of the Hungarian Macrolepidoptera).

[b35] Meurisse N, Hoch G, Schopf A, Battisti A, Gregoire J-C (2012). Low temperature tolerance and starvation ability of the oak processionary moth: implications in a context of increasing epidemics. Agric. For. Entomol.

[b36] Miller DR, Mo TK, Wallner WE (1989). Influence of climate on gypsy-moth defoliation in southern New-England. Environ. Entomol.

[b37] Parmesan C (2006). Ecological and evolutionary responses to recent climate change. Annu. Rev. Ecol. Evol. Syst.

[b38] Parmesan C, Yohe G (2003). A globally coherent fingerprint of climate change impacts across natural systems. Nature.

[b39] Parmesan C, Ryrholm N, Stefanescu C, Hill JK, Thomas CD, Descimon H (1999). Poleward shifts in geographical ranges of butterfly species associated with regional warming. Nature.

[b40] Pierce GJ, Santos MB, Smeenk C, Saveliev A, Zuur AF (2007). Historical trends in the incidence of strandings of sperm whales (*Physeter macrocephalus*) on North Sea coasts: an association with positive temperature anomalies. Fish. Res.

[b41] Pöyry J, Leinonen R, Söderman G, Nieminen M, Heikkinen RK, Carter TR (2011). Climate-induced increase of moth multivoltinism in boreal regions. Glob. Ecol. Biogeogr.

[b42] Robertson C, Nelson TA, Jelinski DE, Wulder MA, Boots B (2009). Spatial-temporal analysis of species range expansion: the case of the mountain pine beetle, *Dendroctonus ponderosae*. J. Biogeogr.

[b43] Saastamoinen M, Vastenhout D, van der Sterren N, Zwaan BJ, Brakefield PM (2010). Predictive adaptive responses: condition-dependent impact of adult nutrition and flight in the tropical butterfly *Bicyclus anynana*. Am. Nat.

[b44] Sinclair RJ, Hughes L (2008). Incidence of leaf mining in different vegetation types across rainfall, canopy cover and latitudinal gradients. Austral Ecol.

[b45] Solomon S, Qin D, Manning M, Chen Z, Marquis M, Averyt KB (2007). Contribution of working group I to the fourth assessment report of the intergovernmental panel on climate change.

[b46] Turchin P (2003). Complex population dynamics: a theoretical/emperical synthesis.

[b47] Turchin P, Taylor AD (1992). Complex dynamics in ecological time series. Ecology.

[b48] Visser ME, Both C (2006). Shifts in phenology due to global climate change: the need for a yardstick. Proc. R. Soc. Lond. B. Biol. Sci.

[b49] Wagenhoff E, Veit H (2011). Five years of continuous *Thaumetopoea processionea* monitoring: tracing population dynamics in an arable landscape of South-Western Germany. Gesunde Pflanz.

[b50] Wallner WE (1987). Factors affecting insect population dynamics: differences between outbreak and non-outbreak species. Annu. Rev. Entomol.

[b51] Walther G-R (2010). Community and ecosystem responses to recent climate change. Philos. Trans. R. Soc. Lond. B. Biol. Sci.

[b52] Walther GR, Post E, Convey P, Menzel A, Parmesan C, Beebee TJC (2002). Ecological responses to recent climate change. Nature.

[b53] West AS (1936). Winter Mortality of Larvae of the European Pine Shoot Moth, *Rhyacionia buoliana* Schiff., in Connecticut. Ann. Entomol. Soc. Am.

[b54] Wood S (2006). Generalized additive models: an introduction with R.

[b55] Zuur AF, Ieno EN, Smith GM (2007). Analysing ecological data.

[b56] Zuur AF, Ieno EN, Walker NJ, Saveliev AA, Smith GM (2009). Mixed effects models and extensions in ecology with R.

